# Genotypes and phenotypes in a *Wolbachia*-ant symbiosis

**DOI:** 10.7717/peerj.17781

**Published:** 2024-07-26

**Authors:** Crystal L. Frost, Rowena Mitchell, Judith Elizabeth Smith, William O.H. Hughes

**Affiliations:** 1 School of Biology, University of Leeds, Leeds, United Kingdom; 2 School of Life Sciences, University of Sussex, Brighton, United Kingdom

**Keywords:** Resistance, Development, Virulence, Caste determination, Social insect, Endosymbiosis

## Abstract

The fitness effects of overt parasites, and host resistance to them, are well documented. Most symbionts, however, are more covert and their interactions with their hosts are less well understood. *Wolbachia*, an intracellular symbiont of insects, is particularly interesting because it is thought to be unaffected by the host immune response and to have fitness effects mostly focussed on sex ratio manipulation. Here, we use quantitative PCR to investigate whether host genotype affects *Wolbachia* infection density in the leaf-cutting ant *Acromyrmex echinatior*, and whether *Wolbachia* infection density may affect host morphology or caste determination. We found significant differences between host colonies in the density of *Wolbachia* infections, and also smaller intracolonial differences in infection density between host patrilines. However, the density of *Wolbachia* infections did not appear to affect the morphology of adult queens or likelihood of ants developing as queens. The results suggest that both host genotype and environment influence the host-*Wolbachia* relationship, but that *Wolbachia* infections carry little or no physiological effect on the development of larvae in this system.

## Introduction

The intracellular bacterium *Wolbachia* is thought to be one of the most abundant endosymbionts on the planet and has been estimated to infect 40–66% of all arthropod species (Hilgenboecker et al., 2008; Zug & Hammerstein, 2012). *Wolbachia* transmits vertically in the maternal line only, resulting in it evolving mechanisms of distorting host sex ratio to increase the proportion or relative fitness of infected female hosts and thereby its own transmission (Werren, 1997). However, *Wolbachia* can also have more classical fitness effects, such as the decrease in longevity seen with the *Wolbachia* strain *w*MelPop in *Drosophila melanogaster* and the enhanced survival seen in the same host when infected with *Wolbachia* strain *w*Dm (Carrington et al., 2009; Fry, Palmer & Rand, 2004). Switching between these phenotypes can occur rapidly, with *Wolbachia* in newly infected populations of *Drosophila simulans* having been shown to evolve from parasitic to mutualistic phenotypes in only 20 years (Weeks et al., 2007). This suggests that the relationship between *Wolbachia* and the biology, and therefore genetic make-up, of its host is important in the effects that *Wolbachia* induces.

The phenotypic effects induced by *Wolbachia* are likely to have a significant impact on the way in which the host immune system interacts with it. While, it was thought that *Wolbachia* can evade detection by the host immune system (Siozios et al., 2008), genetic backcrossing experiments, microinjection experiments, and cycling within natural populations suggest that host physiology affects the success and nature of *Wolbachia* infections (Bordenstein, Uy & Werren, 2003; Evans et al., 2009; Frost et al., 2010; McGraw et al., 2002; Van Borm et al., 2001; Wenseleers, Sundstrom & Billen, 2002). Despite this, research into host genetic effects on *Wolbachia* infection is still limited (Bordenstein, Uy & Werren, 2003; Mouton et al., 2007; Reynolds, Thomson & Hoffman, 2003).

For host resistance traits to spread within a population, resistant individuals must have a selective advantage over non-resistant hosts. One of the most obvious morphological indicators of fitness is size, with larger individuals generally having higher survival and fecundity (Kingsolver & Huey, 2008). Morphological effects of symbionts are well known (Miura et al., 2006), and may also be important in *Wolbachia* infections. If infections cause energetic stress to the host then this may result in larvae developing into smaller adults or adults storing less energy reserves in their fatbody (Arrese & Soulages, 2010; Emlen & Allen, 2003). In support of this, larger mosquitoes have been found to be less likely to pass *Wolbachia* to their offspring, possibly due to these larger females having lower density infections (Kittayapong et al., 2002).

Social insect colonies are made up of reproductive queens and sterile workers, with workers, like males, being an evolutionary dead end for the vertical transmission of *Wolbachia*, potentially selecting for phenotypic effects which increase the representation of *Wolbachia* in queens (termed gynes *sensu stricto* prior to mating). *Wolbachia* infections are common in the ants in particular (Ramalho & Moreau, 2020; Russell et al., 2012; Wenseleers et al., 1998), and in at least one ant species appear to cause sex ratio distortion (Pontieri et al., 2017), but what other fitness effects *Wolbachia* has is unknown. It has been shown in several social insects that patrilines (groups of sisters within a colony that share the same father) may differ in their propensity to develop into different castes, but the reasons for this are largely unknown (Chéron et al., 2011; Holman et al., 2011; Hughes & Boomsma, 2007; Hughes & Boomsma, 2008; Jaffé et al., 2007; Rheindt, Strehl & Gadau, 2005). Symbionts can affect the energy available to hosts for growth and development, so could affect the availability of resources needed to develop as a queen. It is therefore possible that resistance of specific patrilines to *Wolbachia* infection could be the reason for this queen skew, *i.e.* more resistant individuals would have a lower burden of *Wolbachia* infection, leading to less energetic stress and in turn more nutritional resources to develop as queens. If *Wolbachia* exert detrimental effects on their host, resistant patrilines would be expected to have lower density infections and a higher propensity to develop into queens, as these would be overall fitter individuals. The opposite would be the case if *Wolbachia* has beneficial or no effects, as well as if the host has evolved to tolerate *Wolbachia* infections.

*Acromyrmex* leaf-cutting ants have high prevalence *Wolbachia* infections, with infections that appear to cycle within a population over time, suggesting that *Wolbachia* in this group may be subject to significant host regulation (Andersen et al., 2012; Frost et al., 2010; Van Borm et al., 2001). Individual *Acromyrmex* ants may be infected by a single *Wolbachia* strain or multiple coinfecting strains, and *Wolbachia* cells are commonly present in both reproductive and nonreproductive tissues (Andersen et al., 2012; Frost et al., 2014; Sapountzis et al., 2015; Zhukova et al., 2017). Queens of these ants mate with multiple males (Sumner et al., 2004), which facilitates genotypic comparisons because the offspring within a colony share the same environmental conditions, maternal cues and maternal genotype (on average), differing only in their paternal genotype (patriline). This makes *Acromyrmex* ideal candidates for investigation of host effects on *Wolbachia* infection. Here we investigate whether hosts vary genotypically in resistance to *Wolbachia* infection by comparing the *Wolbachia* infection density in queens between patrilines within colonies of *A. echinatior*. We also determine whether any variation in *Wolbachia* density correlates with morphological measures of host fitness or with the propensity of individuals in different patrilines to develop into reproductive queens rather than sterile workers.

## Methods

### Sample collection

We used five monogynous colonies of *Acromyrmex echinatior* (Ae48, Ae357, Ae153, Ae088 and Ae07P4) that were collected in Gamboa, Panama, between 1996 and 2008 (Ae48 in 1996, Ae153 in 2001, Ae07P4 in 2007, and Ae357 and Ae088 in 2008) under permit from the Autoridad Nacional del Ambiente (ANAM), and maintained in the laboratory at 27 ± 2 °C and 80 ± 10% RH on a diet of privet leaves and rice. A total of 96 queens (gynes *sensu stricto*), 96 small workers (<1.2 mm head width) and 96 large workers (1.8–2.4 mm head width) were collected over a week from each colony to allow the queen-worker propensity of each patriline to be determined. All individuals were sampled from the surface of the fungus garden and were of similar age based upon their cuticular colouration (Armitage & Boomsma, 2010). This avoided the propensity estimates being confounded by any temporal changes in sperm use, something which in any case appears to be random in leaf-cutting ants (Holman et al., 2011; Stürup et al., 2014).

### Patriline determination

A single leg per individual was incubated at 56 °C overnight in 100 µl of 5% Chelex 100 (BioRad, Hercules, CA, USA) suspended in 10 µM Tris buffer with 4 µl of Proteinase K (5 µg/ml), and then boiled for 15 min. After spinning down, the DNA extract (supernatant) was removed and used for subsequent PCRs using five microsatellite loci: Ech1390, Ech3385, Ech4126, Ech4225 and Atco15 (see Table 1 for conditions; Helmkampf, Gadau & Feldhaar, 2008; Ortius-Lechner, Gertsch & Boomsma, 2000). An ABI 3130*xl* genetic analyser with Genemapper^®^ v3.7 software was used to score microsatellite sizes in relation to GeneScan^TM^ LIZ-500 size standard (ABI). Individuals were then manually assigned to patrilines based upon their multilocus genotypes, so that queen-worker skew could be measured for each individual patriline. Queen-worker skew was defined as the corrected proportion of females within that patriline that were queens as opposed to workers (the number of queens was corrected by multiplying by two to account for twice the number of workers being collected compared to queens), with <0.5 being worker skewed and >0.5 being queen skewed. Patrilines for which less than seven queens had been sampled were excluded from further analysis.

**Table 1:** Microsatellite PCR conditions. PCR conditions for five microsatellite markers, used to assign parentage in this study. The PCR cycle used was 94 °C for 2 min, 35 cycles of 94 °C for 2 min, annealing temp for 45 s, 72 °C for 2 min, finishing with 7 min at 72 °C.

Primer	Ech1390	Ech3385	Ech4126	Ech4225	Atco15
*Taq* polymerase (Promega) (units/µl)	0.04	0.04	0.04	0.04	0.03
*Taq* buffer	1x	1x	1x	1x	1x
MgCl_2_ (mM)	1.62	0.67	0.67	3.38	2.00
Total dNTPS (µM)	270	100	100	340	200
Primer F (µM)	0.4	0.5	0.5	0.5	0.2
Primer R (µM)	0.4	0.5	0.5	0.5	0.2
Template DNA (µl)	1.1	1.1	1.1	1.1	2
Annealing temperature (°C)	48	50	57	52	55

### *Wolbachia* infection densities

For each queen (only), the density of *Wolbachia* infection was determined using a sample of the ovaries and midgut combined. The tissue samples therefore included both somatic and sexual tissues, as it has been shown that both can harbour substantial densities of *Wolbachia* in these ants (Andersen et al., 2012). Tissues were extracted in the same manner as for genotyping, with the addition of the DNA extract (supernatant) being run through a Onestep-96 PCR inhibitor removal kit (Zymo Research, Tustin, CA, USA). *Wolbachia* was quantified using the comparative Ct method, which corrects for differences in the quantity of host tissue in a sample by combining *Wolbachia* and host DNA quantities into a single value (Schmittgen & Livak, 2008). Host and *Wolbachia* primers and probes were designed using the ABI custom design service based on widely aligned sequences of the relevant *Wolbachia* CoxA gene, designed to detect the multiple *Wolbachia* strains that may infect *Acromrymex* (Andersen et al., 2012; Van Borm et al., 2001), and host 18S rRNA gene (Genbank accession numbers HM211030 and EF012826, respectively). The *Wolbachia* assay included Walter forward primer TTGTATCAACTTTTTCTCACAGACCTGTA, Walter reverse primer AAAAAATGCAGCAGCGT and Walter probe (FAM-NFQ) ACCATAAAGCCAAATACTG. The host assay included Brenda forward primer GCATTCGTATTGCGACGTTAGAG, Brenda reverse primer CATTTTTGGCAAATGCTTTCGCTTC and Brenda probe (FAM-NFQ) ACGATCCAAGAATTTC. 10 µl qPCRs using the Taqman^®^ Universal Master Mix II system were used to determine infection status. Each qPCR contained 1 µl of DNA template, 1× Master mix II, 2.25 mM of each Primer and 0.625 mM of Taqman^®^ probe. The qPCR cycle used was 50 °C for 2 min, 95 °C for 10 min, 40 cycles of 95 °C for 15 s and 60 °C for 1 min. All qPCRs were run in triplicate (average Ct used for analysis), with negative controls and positive reference samples included in each run. Multiple calibration curves were run for both assays. Calibration curves from samples containing ovary and gut tissue had efficiencies of 91–99% for the *Wolbachia* assay and 95–99% for the host assay over 10,000 fold changes for both assays. Additional calibrations were run using whole ants (which had been previously bead-beaten) to extend the calibration curves and these gave similar efficiencies over a greater range (Ct values between 16-37 for the host and 23-36.5 for the *Wolbachia* assay). Any samples which gave Ct values outside of this range were removed from analysis. *Wolbachia* infection density was calculated as the relative quantity (RQ) of *Wolbachia* normalized against the 18S rRNA control gene.

### Morphometric data collection

Morphometric data for queens from four of the five colonies (Ae48, Ae07P4, Ae088 and Ae357) were collected as part of a previous study (Mitchell, Frost & Hughes, 2012). Briefly, six measurements of body size were made for each queen: (1) head width (the maximum head width across the eyes); (2) forewing length (from the first vein intersection to the base of the wing); (3) thorax length; (4) weight (after drying at 70 °C for 5 days). Body parts were scanned (Epson Scan V300 Photo; Epson UK Ltd, Hemel Hempstead, UK) with a resolution of 9600 pixels, and measured using IMAGEJ 1.42q (Rasband 1997–2011) calibrated with a scanned 0.1-mm graticule. Measurement error was estimated as the average coefficient of variation (CV) for each character based on a random subset of 10 individuals measured three times, using Haldane’s correction for small sample size (Haldane, 1955), and was on average <1%.

### Statistical analysis

All analyses were carried out in R version 2.8.1 (R Development Core Team, 2005). The effect of colony on the *Wolbachia* infection density of queens was investigated using a generalised linear model (GLM) with quassi-Poisson error structure and the anova function. The effect of patriline on *Wolbachia* infection density was tested with a linear mixed effects model with patriline nested within colony and both specified as random factors, using the *lmer* function and *lme4* package, with a quasi-Poisson error structure and *p* values computed using the likelihood ratio test method (Bates et al., 2015). The relationships between *Wolbachia* infection density and the various morphological variables on *Wolbachia* infection density were also explored with linear mixed effects models using *lmer*, while the relationship between queen-skew and *Wolbachia* infection density was tested using the *lme* and *ANOVA* functions (Pinheiro et al., 2019). Colony and the interaction between colony and the morphological variable were also tested for in these models. Model choice was determined by examination of residuals.

## Results

**Figure 1:**
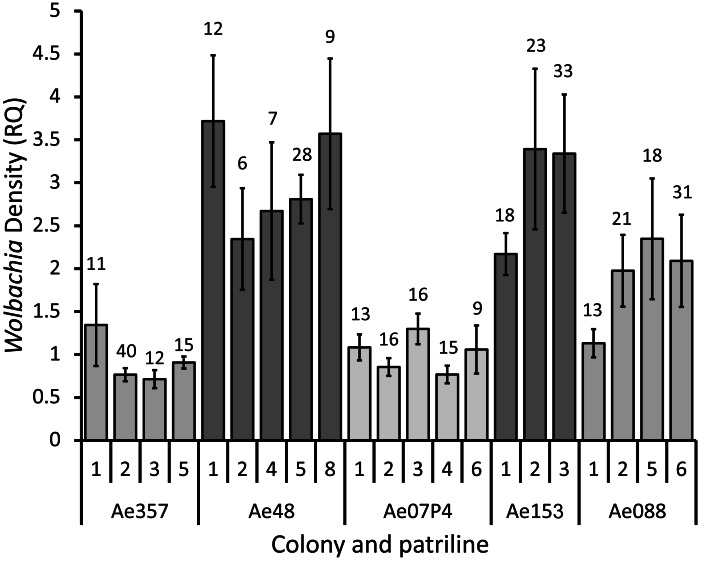
*Wolbachia* infection densities across patrilines. Mean ± SE *Wolbachia* infection density (RQ) for patrilines within five colonies of *Acromyrmex echinatior*. The number of individuals examined for each patriline is shown above the columns. *Wolbachia* infection density refers to the density of *Wolbachia* normalized against the 18S rRNA host control gene.

The density of *Wolbachia* infections in queens (which may be infections by single or multiple strains) differed significantly both between the ant colonies and between patrilines within colonies (respectively: ANOVA (GLM), *f*_360, 4_ = 21.2, *p* < 0.001; LRT (LMER), *f*_24, 20_ = 45.3, *p* = 0.001). The between colony differences were larger than those seen between patrilines within colonies, with colonies Ae48 and Ae153 having infection densities that were 2–4 times as high as colonies Ae357, Ae07P4 and Ae088 (Fig. 1). Colonies with higher density *Wolbachia* infections tended to have greater variation between patrilines, with the largest difference between patrilines being over two-fold (between Patrilines 1 and 5 in colony Ae088; Fig. 1). The more highly infected colonies also appeared to have greater variation within patrilines than the less infected colonies (Fig. 1).

**Figure 2:**
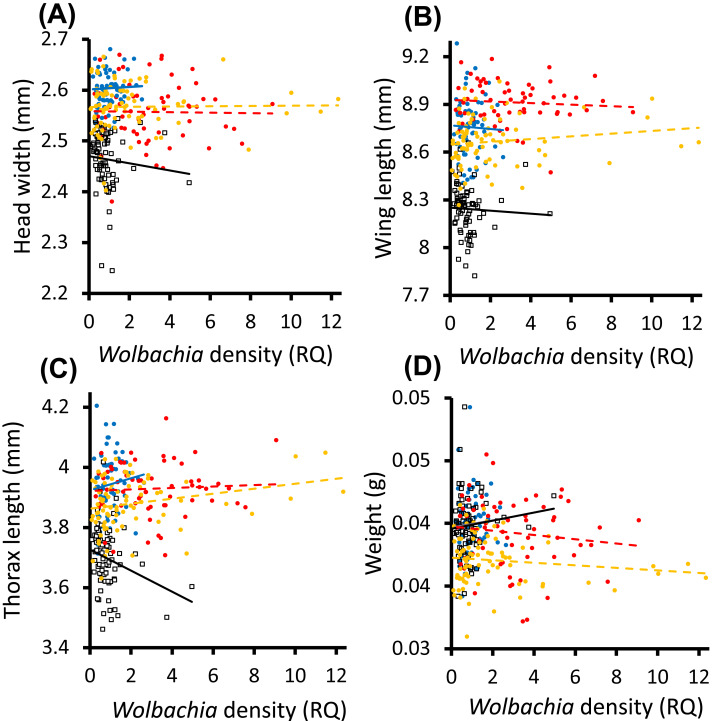
The relationships between *Wolbachia* infection density (RQ) and body size of *Acromyrmex echinatior* queens. Data are for (A) head width, (B) thorax length, (C) wing length and (D) weight for queens from four colonies (colony denoted by different markers: Ae48, red circle and trendline; Ae07P4, blue circle and trendline; Ae088, orange circle and trendline; Ae357, white square and black trendline). *Wolbachia* infection density refers to the density of *Wolbachia* normalized against the 18S rRNA host control gene.

**Figure 3:**
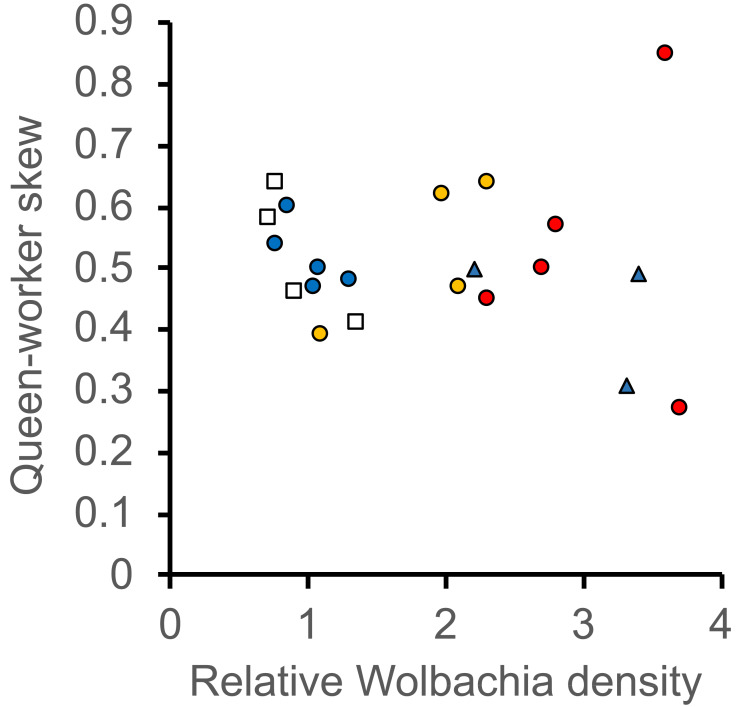
The relationship across leaf-cutting ant patrilines between the density of *Wolbachia* infections (RQ) and the propensity to develop into queens or workers. Caste propensity is queen-worker skew, from 0 being entirely worker-biased to 1 being entirely queen-biased, and 0.5 showing no skew towards either caste. Data are presented for five *Acromyrmex echinatior* colonies indicated with different markers (Ae48, red circle; Ae153, blue triangle; Ae07P4, blue circle; Ae088, orange circle; Ae357, white square). *Wolbachia* infection density refers to the density of *Wolbachia* normalized against the 18S rRNA host control gene.

There was no significant relationship between *Wolbachia* infection density of queens and any of the morphological variables (LRT (LMER), head width, *f*_7, 1_ = 0.036, *p* = 0.849; wing length, *f*_7, 1_ = 5.58, *p* = 0.233; weight, *f*_10, 4_ = 5.796, *p* = 0.215), except for thorax length for which there was a significant interaction between the effects of colony and the density of *Wolbachia* infections on the length of the thorax (LRT (LMER), *f*_10, 4_ = 18.7, *p* < 0.001). Colony Ae357 had a negative relationship between *Wolbachia* infection density and thorax length, while the remaining three colonies had relatively flat relationships (Fig. 2). There was no significant relationship between the infection density and queen-worker skew of patrilines (LME, *f*_15, 1_ = 0.0002, *p* = 0.989, Fig. 3).

## Discussion

There was significant variation among the queens analysed in the density of their *Wolbachia* infections. The greatest differences were between colonies, but there were also statistically significant differences in the infection density of queens between patrilines within colonies. There was a negative relationship between *Wolbachia* infection density and thorax length in one colony, but *Wolbachia* density did not show any significant relationship with morphology in the other colonies, nor the propensity of individuals in a patriline to develop into queens rather than workers. Infections may have involved single or multiple strains because our protocol did not aim to distinguish these.

The largest variation in the *Wolbachia* density of queens was found between colonies, with two colonies (Ae48 and Ae153) having higher density infections than the other three colonies. There is greater genetic variation at this level than within colonies, with individuals across colonies having different maternal, as well as paternal, genes. In addition, environmental effects, epigenetics, the overall health of the colony and differences in the strain composition of *Wolbachia* may also have produced colony-level differences in *Wolbachia* infections. These colony-level differences may therefore be similar to the way populations in other hosts vary in their *Wolbachia* infections due to differences in environmental conditions, genetic composition and symbiont strains (Bordenstein, Uy & Werren, 2003; Fry, Palmer & Rand, 2004; Reynolds, Thomson & Hoffman, 2003). It is interesting to note in this regard that the two colonies with the highest infection densities were the oldest and had been maintained in the laboratory the longest. Laboratory colonies of *Acromyrmex* have previously been found to have higher titres of *Wolbachia* than field colonies (Andersen et al., 2012), and *Wolbachia* can show rapid evolution in response to environmental condition (Weeks et al., 2007). Our results raise the intriguing possibility that this may be cumulative, with the longer a colony is kept in the laboratory under relatively favourable conditions, the greater the infection density. Temperature can have significant effects on *Wolbachia* infection dynamics (Mouton et al., 2007; Pintureau & Bolland, 2001; Reynolds, Thomson & Hoffman, 2003), and it may be that other environmental factors have similar effects. *Wolbachia* in *Acromyrmex* ants is present in the gut and other non-reproductive, as well as reproductive, tissues (Andersen et al., 2012; Frost et al., 2014; Sapountzis et al., 2015; Zhukova et al., 2017), so may show more dynamic infections than endosymbionts that are restricted solely to the ovaries of hosts.

We also found that some patrilines within colonies differed in the density of *Wolbachia* infection of queens. As patrilines within colonies differ only in their paternal genotypes and sperm use in *Acromyrmex* is random (Stürup et al., 2014), this suggests that host genotype can have a significant effect on this endosymbiont. There are three potential explanations for the genotypic variation in infection density. First, that the fitness effects of *Wolbachia* have caused hosts to evolve resistance mechanisms that directly target *Wolbachia* either in general or in a strain-specific way. Second, that *Wolbachia* infection is affected indirectly by immune responses targeted at other threats (Siozios et al., 2008). Third that genotypic differences in some other trait affect *Wolbachia* infection density as an epiphenomenon. The second explanation seems most likely because silkworm cells *in vitro* do not alter gene expression in direct response to *Wolbachia* infection (Nakamura et al., 2011). In addition, the genotypic variation in Wolbachia infection seen here is much lower than the genotypic differences found in resistance to more virulent parasites (Baer & Schmid-Hempel, 2003; Hughes & Boomsma, 2004; Invernizzi, Peñagaricano & Tomasco, 2009). It is important to note that within-colony variation may result from differences in distribution of maternal factors as well as differences between patrilines, but we did not investigate this here.

There were no relationships between *Wolbachia* infection density of queens and any of the morphological variables, with the sole exception of a negative correlation with thorax length in Colony Ae357. The relative lack of *Wolbachia* effects on morphology implies that *Wolbachia* infection density may not be associated with a significant energetic burden or benefit in these hosts, although further experiments would be needed to confirm this by investigating other measures of energetic impact on the host such as metabolic rate (Evans et al., 2009). Comparatively benign infections of many parasites can become more virulent in times of stress (Brown, Schmid-Hempel & Schmid-Hempel, 2003; Comiskey, Lowrie & Wesson, 1999), and it may be that studies looking at other parts of the colony cycle may show a different host-symbiont relationship.

Caste determination in leaf-cutting ants and other social insects is thought to occur through a threshold mechanism whereby environmental cues, such as nutrition, determine the morphological caste into which an individual will develop (Wheeler, 1991). It has been shown in several social insects, including *A. echinatior*, that genotype may affect the propensity of individuals to develop into different castes (Chéron et al., 2011; Holman et al., 2011; Hughes & Boomsma, 2007; Hughes & Boomsma, 2008; Jaffé et al., 2007; Rheindt, Strehl & Gadau, 2005). If *Wolbachia* had an overall positive or negative burden on these insects it may have been expected that genotypic differences in the density of *Wolbachia* infections may correlate with fitness parameters of the host, but we found no evidence to suggest that this is the case. However, it must be noted that colonies varied greatly in their apparent relationships across patrilines between queen-worker skew and the density of *Wolbachia* infection, and the relatively few patrilines studied meant that the analysis had limited power. An analysis which included more colonies and patrilines, with sufficient sample size to provide high statistical power, would be needed to give a more conclusive result.

## Conclusions

Although *Wolbachia* is often considered simply as a maternally transmitted, sex ratio-distorting endosymbiont, there is now abundant evidence that its effects and infection dynamics can be more complex than this. The large differences in infection density between colonies, and smaller differences between patrilines, that we observed here in leaf-cutting ants support this. Endosymbiont infections are unlikely to ever be completely cost-free, and although we found little evidence of any physiological costs or benefits here, further investigation of the physiological impacts of infection would be warranted.

##  Supplemental Information

10.7717/peerj.17781/supp-1 Data S1 Raw Wolbachia density, morphometric, and queen-worker skew data

## Additional Information and Declarations

### Field Study Permissions

The following information was supplied relating to field study approvals (*i.e*., approving body and any reference numbers): Autoridad Nacional del Ambiente (ANAM).

### Data Availability

The following information was supplied regarding data availability: The raw data is available in the Supplemental File.
